# Genomic characterization of DNA damage response pathway mutations reveals their adverse impact on EGFR-TKI efficacy in non-small cell lung cancer

**DOI:** 10.1186/s12931-026-03699-y

**Published:** 2026-05-06

**Authors:** Lujie Yang, Jinli Cheng, Xin Guo, He Xiao, Xunjie Kuang, Yuxin Yang, Lei Zhang, Qin Zhang, Dong Wang, Yu Xu, Mengxia Li

**Affiliations:** 1 Cancer Center, Daping Hospital & Army Medical Center of PLA, Army Medical University, No.10 Changjiangzhi Rd, Yuzhong District, Chongqing, People’s Republic of China; 2 College of Medicine, Chongqing University, Chongqing, China; 3 Department of Radiation Oncology, Sichuan Cancer Hospital & Institute, Sichuan Cancer Center, Cancer Hospital affiliated to School of Medicine, University of Electronic Science and Technology of China,Sichuan, Sichuan, People’s Republic of China; 4 Department of Oncology, Chongqing University Qianjiang Hospital, Chongqing, People’s Republic of China

**Keywords:** EGFR, Non-small cell lung cancer, DNA damage response, Targeted sequencing, Drug resistance

## Abstract

**Background:**

Although tyrosine kinase inhibitors (TKIs) significantly improve survival outcomes in patients with epidermal growth factor receptor (EGFR)-mutant non-small cell lung cancer (NSCLC), tumor clonal evolution under drug pressure inevitably leads to acquired resistance. DNA damage response (DDR) mutations lead to elevated genome instability, but the frequency and impact of DDR gene/pathway mutations on EGFR-TKI treatment efficacy remain unclear.

**Methods:**

A total of 790 NSCLC patients who received high-throughput targeted sequencing at Daping Hospital were retrospectively enrolled to analyze the mutation landscape of DDR genes in NSCLC. Next, 202 EGFR-mutant patients from Daping Hospital and 126 patients from the GENIE database who received first-line EGFR-TKI treatment were further enrolled to explore the association between DDR mutations and clinical outcomes.

**Results:**

DDR genes including ATM, MSH3, BRCA2 (> 5%), and pathways including the Fanconi anemia (FA), homologous recombination repair (HRR), and mismatch repair (MMR) exhibited substantial mutation rates (15%). In the Daping cohort, patients with DDR mutations had inferior outcomes when receiving EGFR-TKI treatment (median PFS: 14.9 vs. 10.8 months, *P* = 0.001) and higher mutation burden. Specifically, FA (HR = 2.314, 95% CI: 1.547–3.549, *P* < 0.001) or HRR (HR = 2.686, 95% CI: 1.786–4.039, *P* < 0.001) pathway mutations were independent risk factors for disease progression. Notably, patients with co-occurring TP53 and DDR mutations had the worst outcomes compared to those with either single mutations or wild-type TP53/DDR, and similar findings were validated in the GENIE cohort.

**Conclusion:**

Our study demonstrates a substantial prevalence of DDR mutations and their association with inferior EGFR-TKI efficacy, highlighting the importance of genomic profiling of DDR mutations to identify this high-risk subpopulation in EGFR-mutant NSCLC.

**Supplementary Information:**

The online version contains supplementary material available at 10.1186/s12931-026-03699-y.

## Background

Epidermal growth factor receptor (EGFR) mutations are one of the principal driver mutations in non-small cell lung cancer (NSCLC) [1, 2]. While EGFR tyrosine kinase inhibitors (TKIs) confer significant clinical benefit and represent the standard first-line therapy for EGFR-mutant NSCLC, acquired resistance inevitably develops in nearly all patients [3–5]. Sequencing of post-treatment specimens has identified mutations associated with acquired resistance, such as EGFR C797S mutation and MET amplification [3, 6, 7]. However, these findings fail to fully explain the heterogeneity of EGFR-TKI efficacy because some patients develop resistance within months, while others can benefit in the long term.

Tumor evolution under the selective pressure of EGFR-TKI therapy is the fundamental mechanism of acquired resistance [8, 9]. Genome instability accelerates this evolutionary process by increasing tumor heterogeneity and promoting the emergence of resistant subclones [10]. Whole genome sequencing has identified more mutation events in the samples after targeted therapy resistance, suggesting that genome instability fuels acquired resistance [11, 12]. The DNA damage response (DDR) system, encompassing multiple pathways safeguarding genome integrity through detecting DNA damage, signaling its presence, and mediating repair, serves as a critical barrier against this instability [13–15]. It is well established that DDR mutations are associated with tumorigenesis and progression [16], and tumors with DDR mutations are linked to accelerated tumor evolution [17]. However, the mutation landscape of DDR genes and pathways, and their impact on EGFR-TKI treatment have not yet been characterized.

Previous studies have identified co-occurring genetic mutations associated with TKI efficacy [18–20]. For example, co-occurring mutations in driver genes or tumor suppressors, such as PTEN, RB1, and TP53, have been associated with poorer clinical outcomes [19]. Notably, the tumor suppressor gene TP53 is the most common co-mutant gene and can be detected in 65% of the EGFR-mutant NSCLC [21]. Multiple studies have reported that TP53 co-occurring mutations is associated with inferior clinical outcomes of EGFR-TKI treatment [22–25]. However, the prognostic impact of TP53 mutation remains inconsistent across studies, particularly in retrospective analyses with limited sample sizes [26, 27], indicating that TP53 status alone may inadequately stratify therapeutic outcomes. As a pivotal effector of the DDR, TP53 preserves genome stability via its transcription factor activity, which orchestrates cell cycle arrest, DNA repair, apoptosis, and cellular senescence [28]. We hypothesize that the consequences of TP53 mutations may be associated with the extent of cellular DNA damage and potentially related to genetic mutations in DDR genes.

In this study, we integrated targeted sequencing and clinical outcomes from two independent cohorts, along with whole exome sequencing (WES) mutation data from The Cancer Genome Atlas (TCGA) database, to profile the mutation landscape of DDR genes in NSCLC. We then evaluated the impact of DDR gene and pathway mutations, and analyzed the effect of TP53 mutation and DDR status on EGFR-TKI treatment efficacy and genome instability.

## Materials and methods

### Patients and sample collections

This study retrospectively included 790 NSCLC patients who received targeted sequencing at Daping Hospital between September 2017 and June 2024. Of them, 368 patients were EGFR-mutant. Within the EGFR-mutant group, we enrolled 202 treatment-naïve patients harboring EGFR-sensitizing mutations who received EGFR-TKIs as first-line treatment for clinical outcome analyses. Of the 202 patients, sequencing specimen types comprised circulating tumor DNA-only (*n* = 71), tissue-only (*n* = 96), and paired circulating tumor DNA and tissue (*n* = 35). Of the 13 patients with stage III disease receiving first-line EGFR-TKI, 5 patients refused chemoradiotherapy; 3 patients had a performance status score > 2; 2 patients had severe renal failure (estimated glomerular filtration rate < 30 mL/min), and 1 patient had severe anemia (Hb < 70 g/L). Due to the immature overall survival (OS) data, only progression-free survival (PFS) was analyzed. The following clinical parameters were collected: age at diagnosis, sex, smoking history, histologic subtype, EGFR mutation subtype, EGFR-TKI generation, and distant metastatic involvement (brain, liver, bone). The TNM staging of the tumor was performed in accordance with the AJCC/UICC 8th edition. The study was approved by the Institutional Review Board of Daping Hospital (No. 2024 − 373), and written informed consent for genomic analysis was obtained from all participants.

### Mutational analyses

Plasma samples and/or formalin-fixed paraffin-embedded tissue specimens were used for targeted next-generation sequencing. Among the 790 patients in the Daping cohort (referred to as the DP cohort), 417 were tissue samples, 184 had circulating tumor DNA samples, and 189 had both sample types. All specimens were subjected to targeted capture sequencing using a 769-gene panel covering 2.4 Mb or an 825-gene panel covering 1.3 Mb of the human genome, aligned to the GRCh37/hg19 reference genome. High-confidence somatic single nucleotide variants (SNVs) and insertions/deletions (indels) were identified and annotated using ANNOVAR, and germline mutations or synonymous mutations were excluded.

### TCGA lung adenocarcinoma (LUAD) and lung squamous cell carcinoma (LUSC) data set

As targeted therapy data are unavailable from the TCGA database, only data from TCGA were used to analyze the mutation frequency of DDR genes and pathways and their association with tumor mutation burden (TMB). Clinical and mutation data of TCGA LUAD and TCGA LUSC cohorts were obtained from cBioPortal (https://www.cbioportal.org/). The TMB_NONSYNONYMOUS value from the “data_clinical_sample” file was directly used to calculate log10-transformed TMB values. The “data_mutations” file was extracted to construct a mutation matrix of DDR genes.

### GENIE cohort data set

Data from the NSCLC cohort version 2.0 from the AACR Project GENIE database were obtained from https://www.synapse.org/ [29]. Clinical data, including sex, age at diagnosis, smoking history, histology, stage, distant metastasis, EGFR-TKI type, treatment line, and survival information, were accessed using synID syn30358089. The sequencing panel information for each patient was obtained via synID syn64501552. The mutation file “data_mutations_extended” was downloaded via synID syn30358120 and used to generate the DDR gene mutation matrix. The “regimen_number” from the Cancer-Directed Regimen Dataset was used to identify patients who received EGFR-TKIs as first-line therapy. PFS was calculated based on “tt_pfs_i_g_mos” as the time interval and “pfs_i_g_status” as the event indicator. OS was calculated based on “tt_os__g_os” as the time interval and “os_g_status” as the event indicator.

### TMB calculation and DDR mutation status

TMB was calculated using nonsynonymous mutations, including missense, nonsense and indels divided by the total size of 2.4 Mb human genome regions covered by 769-gene panel or 1.3 Mb human genome regions by 825-gene panel. In order to normalize panel-specific TMB distributions for DP cohort, the power transformation followed by standardization of z scores was used according to the method proposed in the article [30]. The formulas for power transformation were (TMB)^0.15^ and − 1×(TMB)^(−0.05)^ for 769-gene panel and 825-gene panel respectively. These formulas were obtained through function transformTukey implemented in R package companion (version 2.5.1). The same method was applied for TMB calculation for GENIE cohort except for the total size covered by individual panel which was obtained from the article [30]. DDR genes and pathways listed in supplementary Table 1 were constructed based on a comprehensive review of articles about DDR published before [31, 32]. Patients were classified as DDR mutation with at least one DDR gene or pathways mutation. For the analysis of mutations, germline mutations and synonymous mutations were excluded, and no functional annotation was performed.

### Statistical analysis

The difference in TMB was assessed with Kruskal-Wallis test accompanied by stepwise comparisons with Bonferroni Correction for multiple comparisons among DDR gene mutation groups [wild-type (WT) vs. mutation] or with Wilcoxon rank-sum test between WT and mutation groups for FA and HRR pathways. Differences in baseline clinical characteristics between the DP cohort and GENIE cohorts as well as difference in distant metastasis rate or groups and number of metastatic organs among DDR gene mutation groups, were examined using the Chi-square test or Fisher’s exact test. Univariate Cox regression was used to evaluate the prognostic significance of clinical factors and DDR gene mutations (WT vs. mutation) or DDR gene mutation at individual gene level or at pathway level. The DDR genes or pathways with at least 5 patients carrying mutations were included in analyses in each cohort. Clinical factors adjusted in the multivariate Cox proportional hazards regression included sex (male vs. female), age (≥ 65 vs. < 65 years), smoking history (yes vs. no), stage (IV vs. IIIA-IIIC), EGFR mutation type (L858R/others vs. exon 19 deletion), EGFR-TKI (third vs. first/second generation) and brain metastasis (yes vs. no) in the DP cohort. Only smoking history (yes vs. no), EGFR mutation type (L858R vs. exon 19 deletion, T790M/others vs. exon 19 deletion) and EGFR-TKI (second generation vs. first generation, third generation vs. first generation) in the GENIE cohort were adjusted in the model to mitigate overfitting, as these three clinical factors were shown to be significant in the univariate Cox regression. Analyses for association between PFS and mutations at gene-level were exploratory and not adjusted for multiple comparisons. Kaplan-Meier curves and log-rank test were used to compare PFS for different groups in the univariate setting. In order to more clearly illustrate the differences in PFS in multivariate setting, hazard-adjusted survival curves and restricted mean survival time (RMST) were also compared. In addition, sensitivity analyses were used to further explore the independent prognostic impact of FA/HRR pathway mutation after adjusting for mutations in other DDR genes or major pathways. Survival analyses were performed using R package survival (version 3.8.3) and adjustedCurves (version 0.11.3). The HRs and 95% confidence interval (CI) of DDR genes from Cox regression were visualized using forest plots. All reported P values were two-sided, with statistically significant defined as a two-sided P value < 0.05. All analyses were performed using R software version 4.2.3.

## Results

### Patient characteristics

This retrospective study included two independent cohorts. A total of 790 lung cancer patients who received targeted sequencing covering 48 DDR pathway-related genes at Daping Hospital were analyzed. Among these patients, 422 (53.4%) were EGFR-WT, and 368 (46.6%) were EGFR-mutant. Within the EGFR-mutant group, 202 EGFR-sensitizing mutant patients receiving first-line EGFR-TKI treatment with complete clinical records were included in the survival analyses. Additionally, the GENIE cohort provided mutational data from 139 EGFR-sensitizing mutation NSCLC patients treated with first-line EGFR-TKIs, and 126 of them had available PFS follow-up data. The DDR genes and pathways analyzed in both the DP and GENIE cohorts were listed in Supplementary Table 1.

Table 1 summarizes the baseline characteristics of patients prior to treatment. In the DP cohort, most patients were non-smokers (75.74%) and female (59.41%), and nearly all patients had lung adenocarcinoma (97.52%). EGFR mutations included exon 19 deletion (19del) in 96 patients (47.52%) and L858R substitutions in 93 patients (46.04%). Regarding first-line TKI therapy, 125 patients (61.88%) received first-generation TKIs (icotinib, erlotinib, or gefitinib), 60 (29.70%) received third-generation TKIs (almonertinib or osimertinib), and 17 patients (8.42%) received second-generation TKIs (afatinib). Approximately 70% of patients presented with distant metastasis, most commonly in the bones (55.45%) and brain (35.64%). Regarding DDR mutational status, 82 patients (40.59%) had at least one DDR gene mutation, while 120 patients (59.41%) were DDR WT. In the GENIE cohort, only 33 patients (23.74%) harbored DDR gene mutations, which was attributed to the limited DDR gene panel (17 genes). Other clinical characteristics of the GENIE cohort are summarized in Table 1.

**Table 1:** Clinical characteristics of the Daping and GENIE cohorts

Characteristics		Daping cohort *n* = 202(%)	GENIE cohort *n* = 139(%)
Sex	Female	120 (59.41%)	96 (69.06%)
	Male	82 (40.59%)	43 (30.94%)
Age	> 65	61 (30.20%)	49 (35.25%)
	≤ 65	141 (69.80%)	90 (64.75%)
Smoking	No	153 (75.74%)	80 (57.55%)
	Yes	49 (24.26%)	59 (42.45%)
Histology	Adenocarcinoma	197 (97.52%)	112 (80.58%)
	Squamous cell carcinoma	5 (2.48%)	19 (13.67%)
Stage	I-II	0	19 (13.67%)
Stage III	IIIA	2 (0.99%)	9 (6.47%)
	IIIB	7 (3.47%)	
	IIIC	2 (0.99%)	
Stage IV	IVA	50 (24.75%)	111 (79.86%)
	IVB	141(69.81%)	
EGFR mutation	Exon 19 deletion	96 (47.52%)	67 (48.20%)
	L858R	93 (46.04%)	64 (46.04%)
	L861Q	7 (3.47%)	1(0.72%)
	L719X	2 (0.99%)	1(0.72%)
	Others	4 (1.98%)	6 (4.32%)
TKI generation	First generation	125 (61.88%)	114 (80.58%)
	Second generation	17 (8.42%)	8 (5.76%)
	Third generation	60 (29.70%)	17 (12.23%)
Metastases(sites)	No	179 (88.61%)	90 (64.75%)
	Liver	23 (11.39%)	49 (35.25%)
	No	130 (64.36%)	53 (38.13%)
	Brain	72 (35.64%)	86 (61.87%)
	No	90 (44.55%)	42 (30.22%)
	Bone	112 (55.45%)	97 (69.78%)
DDR mutation status	Mutation	82 (40.59%)	33 (23.74%)
	Wild type	120 (59.41%)	106 (76.26%)

### Mutational Landscape of DDR Genes and Pathways in EGFR-Mutant NSCLC

To comprehensively investigate the mutation frequency of DDR genes and pathways in EGFR-mutant lung cancer, we first analyzed 89 EGFR-mutant lung cancer patients from the TCGA database with available WES data. DDR genes including FANCM, ATM, EXO1, PARP3, POLE, BRCA1, BRCA2, BRIP1, and HFM1 showed considerable mutation rates (4%–8%) (Supplementary Fig. 1A). The proportion of DDR co-occurring mutations in EGFR-mutant NSCLC was 67.4%, which was lower than that in EGFR-WT group (78.2%). For DDR pathways, homologous recombination repair (HRR), nucleotide excision repair (NER), cell-cycle checkpoint factors (CPF), Fanconi anemia (FA), base excision repair (BER) and mismatch repair (MMR) pathways also demonstrated high mutation frequencies (> 20%; Supplementary Fig. 1C).

**Fig. 1:**
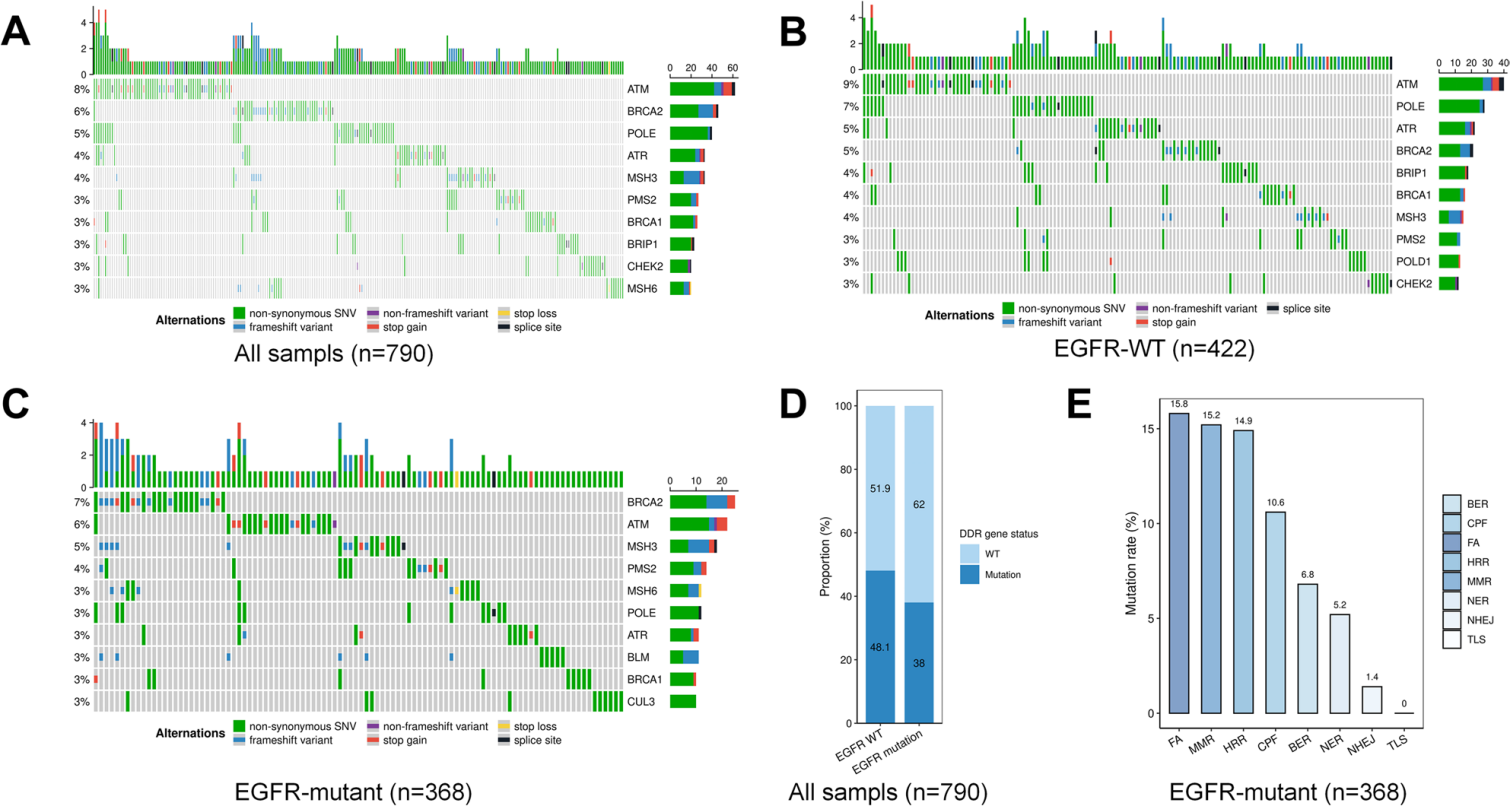
Profiling the DDR gene and pathway mutation landscape of NSCLC in the DP cohort. **A** Oncoprint showing the mutation status of the top 10 DDR genes in the DP cohort (*n* = 790).(B-C)Oncoprint showing the mutation status of the top 10 DDR genes in EGFR-WT (*n* = 422) (**B**) and EGFR-mutant (*n* = 368) (**C**) NSCLC in the DP cohort. **D **Proportion of NSCLC with DDR mutations in EGFR mutation or WT groups (*n* = 790). **E** Mutation frequency of DDR pathways in EGFR-mutant NSCLC in the DP cohort (*n* = 368)

We next validated these findings in the DP cohort. Among all 790 NSCLC patients, 1112 DDR mutations were detected, of which 792 were non-synonymous SNV (71.2%) and 301 truncating mutations (27.1%). The top ten mutated DDR genes—ATM, BRCA2, POLE, ATR, MSH3, PMS2, BRCA1, BRIP1, CHEK2 and MSH6—exhibited mutation frequencies ranging from 3% to 8% (Fig. 1A). Comparative analysis between EGFR-mutant and EGFR-WT patients revealed similar DDR gene mutation profiles (Fig. 1B, C). Consistent with the results in TCGA, EGFR-WT NSCLC had a higher proportion of DDR gene mutations than EGFR-mutant NSCLC (48.1% vs. 38%). Within the EGFR-mutant group, FA, HRR and MMR pathways showed substantial mutation frequencies (> 15%), followed by CPF (10.6%) (Fig. 1D).

Validation in the GENIE cohort identified 13 DDR genes with at least one mutation. ATM, BRCA1, NBN, PALB2, and BRCA2 showed mutation frequencies exceeding 3% (Supplementary Fig. 1C).

### DDR co-occurring DDR mutations and FA/HRR pathway mutations are independent risk factors associated with disease progression

**Fig. 2:**
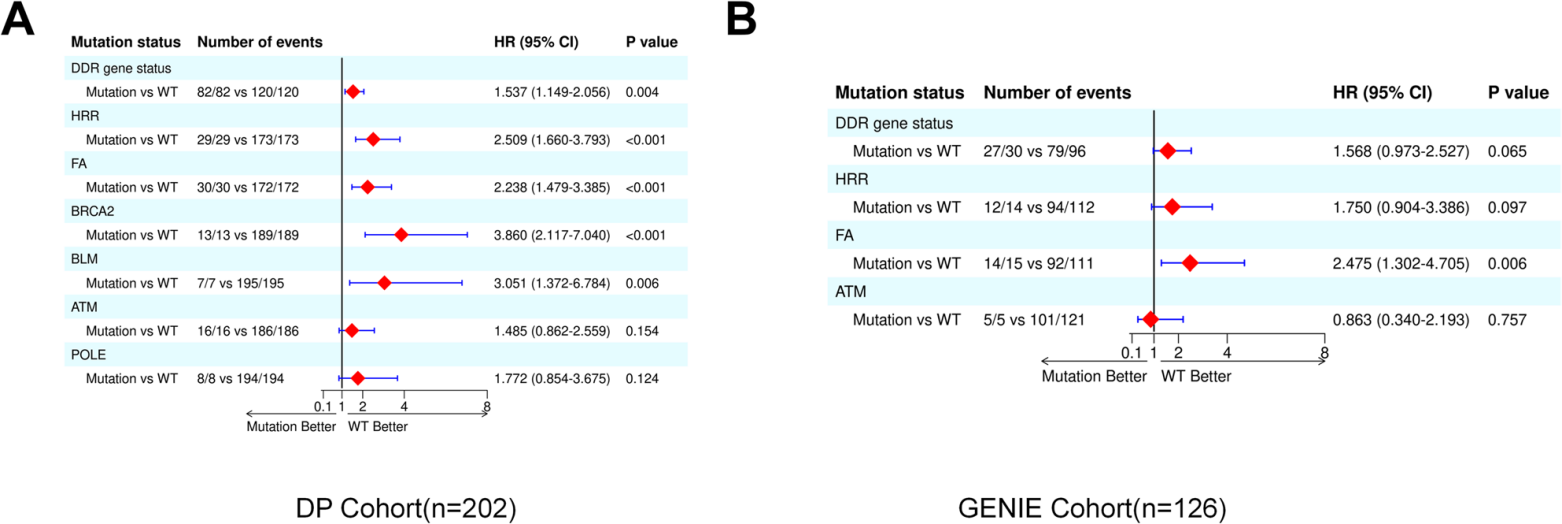
Multivariate Cox analyses for PFS in the DP and GENIE cohorts. **A **Multivariate analyses for PFS in the DP cohort after adjustment for factors including sex, age, smoking history, tumor stage, EGFR mutation type and EGFR-TKI and brain metastasis. **B **Multivariate analyses for PFS in the GENIE cohort after adjustment for clinical factors including smoking, EGFR mutation type and EGFR-TKI. P values were derived from Wald tests

We then evaluated the impact of DDR co-occurring mutations and specific DDR gene or pathway mutations on disease progression. Univariate Cox regression identified DDR gene mutations as a significant predictor of shorter PFS (HR = 1.608, 95%CI: 1.206–2.146, *P* = 0.001) (Supplementary Fig. 2A). Specifically, BRCA2, POLE, ATM and BLM genes, as well as mutations in the FA and HRR pathways were independent risk factors for disease progression (Supplementary Fig. 2A). DDR mutations remained statistically significant in multivariate analyses after adjusting for clinical covariates (HR = 1.537, 95%CI: 1.149–2.056, *P* = 0.004) (Fig. 2A). Additionally, mutations in BRCA2, BLM, as well as mutations in the FA (HR = 2.238, 95%CI: 1.479–3.385, *P* < 0.001) and HRR (HR = 2.509, 95%CI: 1.660–3.793, *P* < 0.001) pathways, remained independently associated with poorer PFS (Fig. 2A).

Validation in the GENIE cohort confirmed that DDR gene mutations independently predicted poorer PFS (HR = 1.779, 95% CI: 1.125–2.812, *P* = 0.002) by univariate analysis (Supplementary Fig. 2B). However, only FA mutations (HR = 2.475, 95%CI: 1.302–4.705) remained significantly associated with disease progression after adjustment for smoking, EGFR mutation type and EGFR-TKI (Fig. 2B). The results of univariate Cox regression analyses for all clinical factors and DDR gene or pathway mutations for the DP cohort and GENIE cohort were summarized in Supplementary Table 2.

### DDR co-occurring mutations are related to worse clinical outcomes and increased TMB in EGFR-mutant NSCLC

**Fig. 3:**
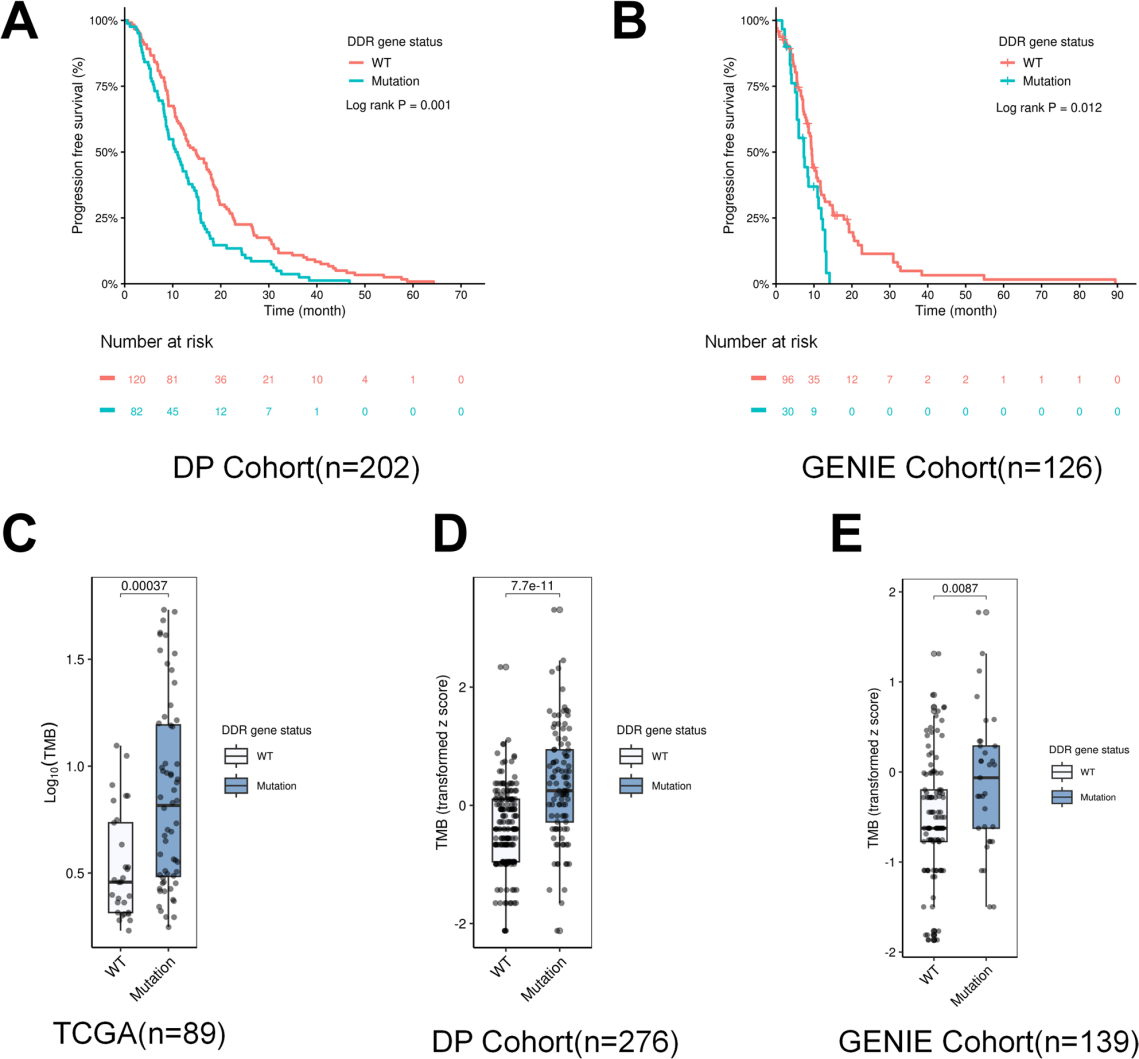
DDR mutations are related to shorter PFS and increased TMB in the DP and GENIE cohorts. EGFR-mutant NSCLC patients were divided into two groups based on DDR mutation status. **A-B** Kaplan-Meier plots showing patients harboring DDR mutations had significantly worse PFS than DDR WT in the DP cohort (*n* = 202) **A** and GENIE cohort (*n* = 126) **B**. **C-E **TMB of patients with DDR mutation or DDR WT in TCGA **C**, DP **D** and GENIE **E** cohorts. The *P* values were assessed by the log-rank test in **A-B** and Wilcoxon rank-sum test in **C-E**

Next, we evaluated the survival of EGFR-mutant patients with or without DDR mutations. Baseline characteristics were well-balanced between the two groups, except for a slightly lower proportion of 19del in the DDR-mutant group compared to the DDR-WT group (36.59% vs. 42.5%) (Supplementary Table 3). As expected, EGFR-mutant patients with DDR mutations had shorter PFS in both the DP (mPFS: 14.9 vs. 10.8 months, log-rank *P* = 0.001) and GENIE cohorts (mPFS: 9.44 vs. 7.34 months, log-rank *P* = 0.01) (Fig. 3A–B). The RMST in patients harboring DDR gene mutations was significantly shorter than those without DDR mutations [RMST: 12.79 (95% CI: 10.50-15.09) vs. 17.88 months (95% CI: 15.98–19.78), Z test *P* < 0.001]. The significantly shorter RMST in patients with DDR gene mutations was also observed in the GENIE cohort [RMST: 8.23 (95% CI: 6.83–9.63) vs. 13.35 months (95% CI: 9.91–16.79), Z test *P* = 0.006] (Supplementary Fig. 3A, B).

We hypothesized that the shortened PFS might be related to increased genome instability. To evaluate genome instability of samples, we analyzed TMB in the TCGA, DP and GENIE cohorts. The harmonization of crude TMB values with power and z transformations yielded adequate concordance of value distribution across panels used in both the DP and GENIE cohorts, making direct comparisons feasible within each cohort. Tumors with DDR gene mutations exhibited significantly higher TMB compared to DDR-WT tumors in all three cohorts (all *P* < 0.05), indicating the increased genome instability in EGFR-mutant NSCLC with co-occurring DDR mutation (Fig. 3C-E). The statistical performance of crude and transformed TMB values in both cohorts was summarized in Supplementary File1.

### DDR co-occurring mutations correlate with an aggressive metastatic phenotype

Metastatic spread is a major contributor to cancer-related mortality. We analyzed the metastases of patients, and found that patients with DDR gene mutations exhibited higher rates of metastatic events. Specifically, patients with DDR mutations showed numerically higher rates of bone metastases (58.5% vs. 53.3%, *P* = 0.557), brain metastases (41.5% vs. 31.7%, *P* = 0.201) and liver metastases (14.6% vs. 9.2%, *P* = 0.329) without statistical significance in the DP cohort. For the GENIE cohort, patients with DDR mutations also showed a higher proportion of liver metastases (40.7% vs. 31.6%, *P* = 0.531), head and neck metastases (48.1% vs. 27.8%, *P* = 0.089), suggesting that DDR co-occurring mutations may be associated with a more aggressive metastatic phenotype (Supplementary Fig. 3C, D).

### FA/HRR pathway mutations are related to markedly poorer clinical outcomes and higher genome instability

**Fig. 4:**
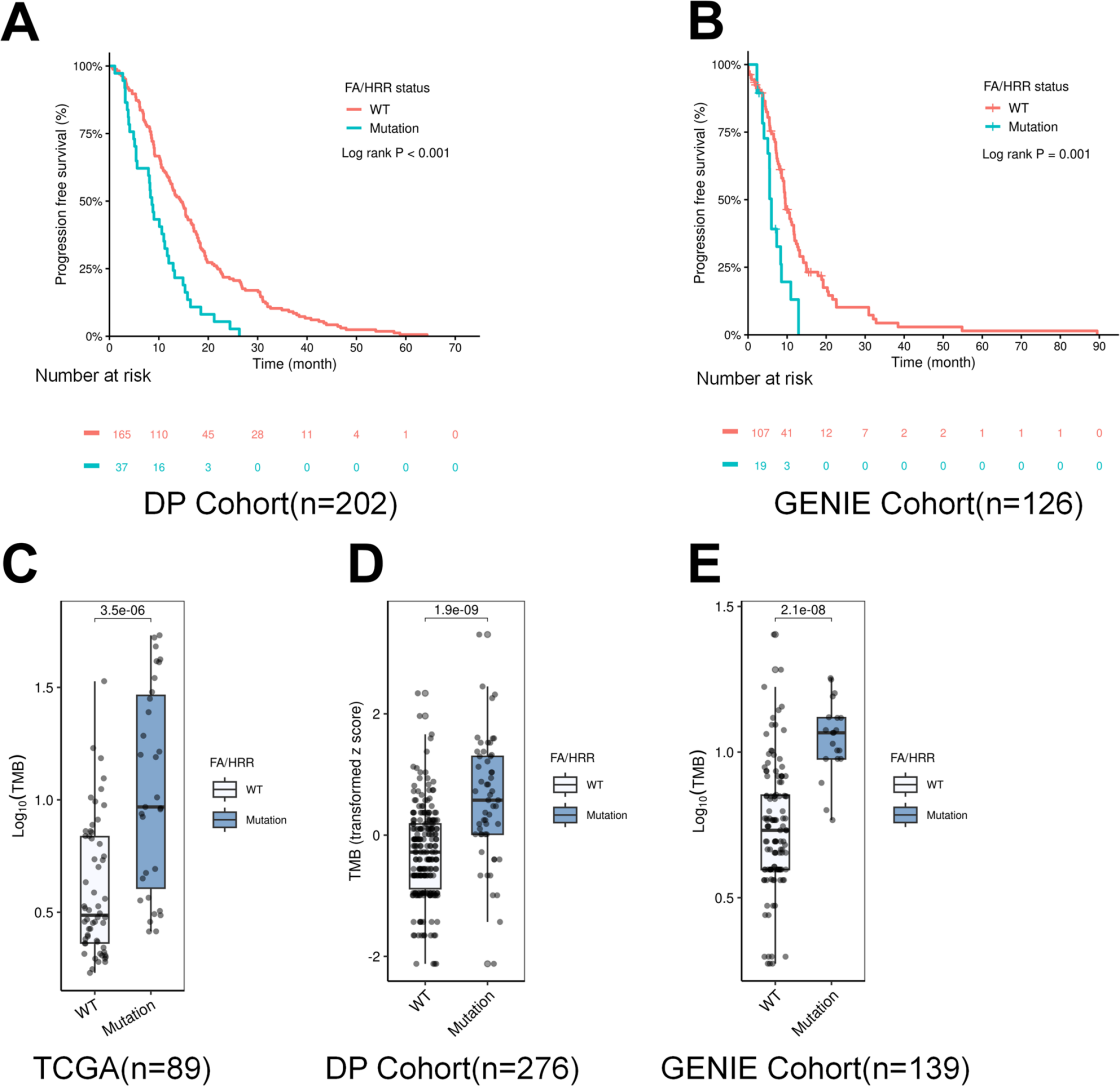
Co-occurrence of FA/HRR pathway mutations is associated with markedly poorer PFS and higher TMB in the DP and GENIE cohorts. EGFR-mutant NSCLC patients were divided into two groups based on FA/HRR pathways status. **A-B** Kaplan-Meier plots showing the PFS of EGFR-sensitizing mutant NSCLC with or without FA/HRR mutations in the DP cohort (n = 202) (**A**) and GENIE cohort (n = 126) (**B**). **C-E** TMB of patients with or without FA/HRR mutations in the TCGA (**C**), DP (**D**) and GENIE (**E**) cohorts. The *P* values were assessed by the log-rank test in **A-B** and Wilcoxon rank-sum test in **C-E**

Because FA and HRR pathway mutations were independent risk factors for disease progression, we compared clinical outcomes between patients with or without FA/HRR pathway mutations. Due to the overlap of genes in the FA and HRR pathways, patients with FA or HRR mutations were combined into a single group. The results showed that EGFR-mutant NSCLC with FA/HRR mutations had a markedly shortened PFS in both the DP (mPFS: 14.5 vs. 8.6 months, log-rank *P* < 0.001) and GENIE cohorts (mPFS: 9.54 vs. 5.99 months, log-rank *P* < 0.001) (Fig. 4A, B). The RMST in the patients with FA/HRR mutations was significantly shorter than that in FA/HRR WT patients as revealed by confounder-adjusted survival curve analyses in the DP cohort [RMST: 9.78 (95% CI: 7.59–11.97) vs. 17.08 months (95% CI: 15.49–18.68), Z test *P* < 0.001]. A significantly shorter RMST was also observed in the GENIE cohort [RMST: 6.98 (95% CI: 5.68–8.28) vs. 12.51 months (95% CI: 9.85–15.17), Z test *P* < 0.001] (Supplementary Fig. 4A, B). Multivariate Cox regression results are presented in Supplementary Table 4. Sensitivity analyses also showed that the HR for FA/HRR mutations was not dramatically changed after adjustment for other common mutated DDR genes and non-FA/HRR pathways in both cohorts (Supplementary Fig. 5A, B) (Supplementary Table 5). Additionally, TMB was significantly higher in patients with FA/HRR pathway mutations in both the DP and GENIE cohorts (Fig. 4C-E).

### TP53 mutations have comparable and cumulative effects with DDR mutations on genome instability and EGFR-TKI efficacy

**Fig. 5:**
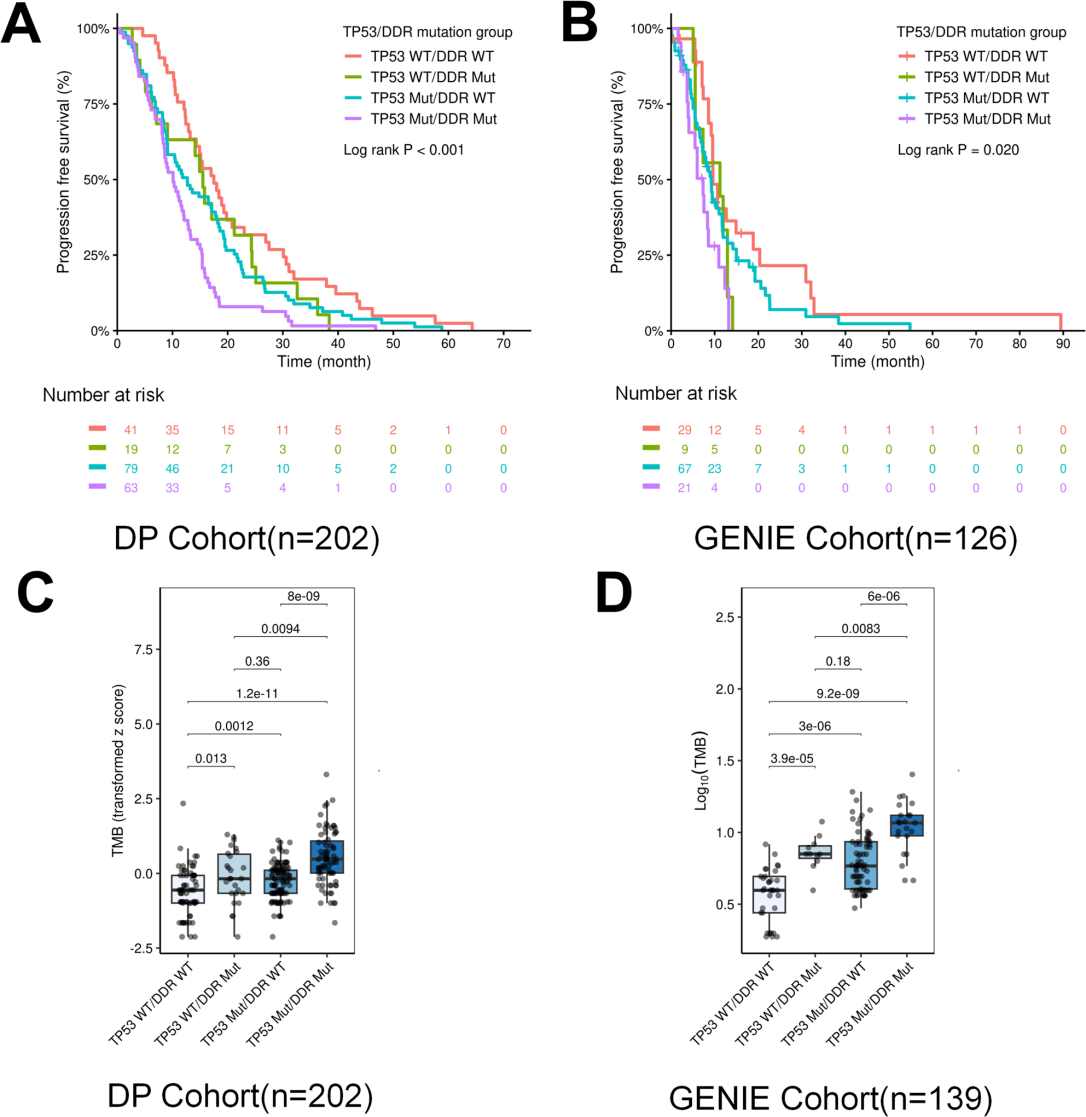
Combination of TP53 mutation and DDR status to differentiate survival and TMB in EGFR-mutant NSCLC. EGFR-mutant NSCLC patients were divided into four groups based on TP53 and DDR mutation status. **A-B** Kaplan-Meier plots of EGFR-mutant NSCLC with TP53 WT/DDR WT, TP53 mutation/DDR WT, TP53 WT/DDR mutation or TP53 mutation/DDR mutation in the DP cohort (n = 202) (**A**) and GENIE cohort (n = 126) (**B**). **C-D **TMB of patients with EGFR-mutant NSCLC with TP53 WT/DDR WT, TP53 mutation/DDR WT, TP53 WT/DDR mutation or TP53 mutation/DDR mutation in the DP (**C**) and GENIE (**D**) cohorts. The P values were assessed by the log-rank test in **A-B** and Wilcoxon rank-sum test in **C-D**

Though TP53 is not a typical DDR kinase, it plays an important role in responding to DNA damage as a transcription factor. We hypothesized that the impact of TP53 mutation on EGFR-TKI efficacy is related to DDR status. To further explore the role of TP53 mutations, we categorized patients into four groups based on TP53 and DDR gene mutation status: TP53 WT/DDR WT, TP53 WT/DDR mutation, TP53 mutation/DDR WT, and TP53 mutation/DDR mutation. The results showed that the TP53 mutation/DDR mutation group had the worst PFS, and the TP53 WT/DDR WT group had the best PFS (log-rank adjusted *P* < 0.001) (Fig. 5A, B). In the DDR WT subgroup, patients with TP53 mutation had shorter PFS compared with TP53 WT (mPFS: 12.7 vs. 17.5 months, log-rank adjusted *P* = 0.068). A similar trend was also observed in the subset of DDR mutation group: patients with TP53 mutation had shorter PFS compared with TP53 WT (mPFS: 10.1 vs. 15.5 months, log-rank adjusted *P* = 0.070). Cox regression with TP53, DDR and interaction term showed that the interaction was not significant (P_interaction_ = 0.687). The interaction term was also not statistically significant in the GENIE cohort (P_interaction_ = 0.995), indicating that mutations in TP53 and DDR mutations exerted a cumulative rather than synergistic effect.

In line with the survival outcomes, patients with co-occurring TP53 mutation/DDR mutation had the highest TMB among the four groups. Furthermore, no significant difference was observed between TP53 WT/DDR mutation and TP53 mutation/DDR WT groups (Fig. 5C, D).

## Discussion

In this study, we comprehensively characterized the mutational landscape of DDR genes in EGFR-mutant NSCLC and identified frequent mutations in DDR genes, including BRCA2 (7%), ATM (6%) and MSH3 (5%), as well as in DDR pathways such as FA (15.8%) and HRR (15.2%). Furthermore, we found that patients with DDR co-occurring mutations exhibited significantly worse PFS and higher genome instability, particularly in those with FA and HRR pathways mutations. Finally, we explored the comparable and cumulative effects of TP53 mutation with DDR status on EGFR-TKI efficacy.

DDR is strongly associated with genome stability and treatment response, while previous studies have largely focused on the role of DDR gene mutations as targets and biomarkers for immunotherapy [31, 33]. Our study analyzed the association of DDR mutations with targeted therapy efficacy in two cohorts, and demonstrated that BRCA1, BRCA2, BLM mutations and FA/HRR pathway mutations correlate with poor prognosis. Unlike earlier reports suggesting mutual exclusivity between BRCA2 and EGFR mutations in the MSK-IMPACT platform [25], we observed comparable BRCA1/2 mutation frequencies in both EGFR-mutant and EGFR-WT patients, indicating the possible ethnic variability or cohort differences in the Chinese population. The established synthetic lethality between BRCA mutations and poly ADP-ribose polymerase (PARP) inhibition in breast cancer offers a compelling therapeutic strategy. Notably, a case report documented sustained response to olaparib in a BRCA1-mutant, EGFR-TKI-resistant NSCLC patient [34]. Our study observed that lung cancer patients with co-occurring mutations in the FA/HRR pathway exhibited markedly poorer benefit from TKI therapy. We hypothesized that combination therapy with PARP inhibitors could potentially improve survival outcomes in these patients; however, well-designed prospective clinical trials are needed to validate this hypothesis.

TMB is an important indicator reflecting genome instability [35]. Previous studies have shown that TMB obtained from different targeted sequencing panels exhibit a robust Pearson correlation with those from WES (*R* > 0.9) [36, 37]. Our results demonstrate that DDR-mutant NSCLC exhibits elevated TMB, supporting the concept that impaired DDR accelerates clonal evolution under the selective pressure of EGFR-TKI treatment. This aligns with observations linking elevated TMB to inferior outcomes in EGFR-mutant NSCLC [22]. These findings highlight the potential necessity of expanded sequencing panels to more accurately predict EGFR-TKI efficacy. However, the use of different sequencing panels and sample types in our study may introduce bias in the calculation of mutation frequency and TMB. While sequencing results from ctDNA demonstrated acceptable concordance with those from tissue specimens [38], the imbalance in sample types unavoidably introduced bias into mutation detection and TMB calculation. Moreover, varied panel size, capture regions and sequencing depth in different panels also cause inconsistent variant calling. These factors may affect the accuracy of the results. In addition, we did not categorize DDR mutations using strict functional annotation in this study because of the incomplete mutation annotation currently available. It should be noted that only a proportion of missense mutations are functionally impactful [39], highlighting that our findings reflect the correlation between DDR mutation burden (rather than DDR functional mutations) and the efficacy of EGFR-TKIs. In summary, the proposition that DDR mutations drive tumor clonal evolution under TKI therapeutic pressure remains theoretical, and basic research and multi-omic profiling of clinical specimens will be required in the future.

Encouragingly, two randomized phase III trials including NEJ009 and FLAURA2 have demonstrated the superiority of upfront combination with chemotherapy over EGFR-TKI monotherapy in advanced EGFR-mutant NSCLC [40, 41]. However, the subgroup analysis failed to identify which patients would benefit more from combination therapy versus TKI monotherapy based on clinical characteristics or sensitizing EGFR variants. Our study observed the poorer PFS in DDR-mutant patients, particularly those with FA/HRR mutations, defining a high-risk subpopulation for whom EGFR-TKI monotherapy may be inadequate. Moreover, prior studies have shown that DDR-mutant, driver gene WT NSCLC patients had longer PFS when receiving first-line platinum-based chemotherapy, indicating the potential sensitivity to chemotherapy in DDR-mutant lung cancer [32]. Therefore, combining EGFR-TKIs with chemotherapy may provide clinical benefit for this high-risk subpopulation, highlighting the need for prospective clinical trials to validate this hypothesis.

Our study has inherent limitations: (1) targeted sequencing included only 48 (DP cohort) or a restricted 18-gene DDR panel (GENIE cohort) rather than whole-genome sequencing, which may underestimate the full spectrum of DDR mutations (e.g., omission of FANCM, which is frequently mutated in TCGA). This limitation led to significant discrepancies in mutation frequencies between cohorts, indicating that the pathway-level reproducibility is more reliable than individual gene-level comparisons (2). The retrospective design introduces potential biases in outcome assessment, and heterogeneity in first-line TKI generations across patients may confound the survival analyses. In particular, imbalances in sample types and sequencing panels in the DP cohort may lead to inaccuracies in mutation detection and TMB calculation. (3, 4) No central pathology review was conducted in our study, and investigations into post-resistance treatments and resistance mechanisms remain limited.

## Conclusions

Our study delineated the mutational landscape of DDR genes and pathways in EGFR-mutant NSCLC, and demonstrated that co-occurring mutations in DDR genes are associated with worse survival outcomes and increased genome instability. We further revealed the comparable and cumulative effects of TP53 mutations with DDR mutations on EGFR-TKI efficacy. These findings underscore the importance of genomic profiling for DDR mutations, and provide insights into the potential treatment strategies of combination therapies in EGFR-mutant NSCLC with DDR mutations. Our findings are prognostic and hypothesis-generating, indicating that basic research and prospective clinical studies are needed to further validate the mechanistic link between specific DDR functional mutations and tumor evolution under EGFR-TKI treatment.

## Supplementary Information

Supplementary Material 1.

Supplementary Material 2.

Supplementary Material 3.

Supplementary Material 4.

Supplementary Material 5.

Supplementary Material 6.

## Data Availability

The data supporting the findings of this study are restricted by the Ethics Committee of Daping hospital due to privacy and ethical concerns. Requests for access to these data can be made to the corresponding author and the Ethics Committee with a detailed research proposal.

## Abbreviations

EGFR: Epidermal growth factor receptor
TKIs: Tyrosine kinase inhibitors
NSCLC: Non-small cell lung cancer
DDR: DNA damage response
TCGA: The Cancer Genome Atlas
OS: Overall survival
PFS: Progression-free survival
TMB: Tumor mutation burden
HRR: Homologous recombination repair
NER: Nucleotide excision repair
CPF: Cell-cycle checkpoint factors
FA: Fanconi anemia
BER: Base excision repair
MMR: Mismatch repair
